# Multiple autoantibody-producing B-cell clones coexist within a single human salivary gland

**DOI:** 10.1093/rheumatology/keag525

**Published:** 2026-09-18

**Authors:** Masaru Takeshita, Katsuya Suzuki, Kazuyuki Tsunoda, Satoshi Usuda, Yuko Kaneko

**Affiliations:** Division of Rheumatology, Department of Internal Medicine, Keio University School of Medicine, Tokyo, Japan; Division of Rheumatology, Department of Internal Medicine, Keio University School of Medicine, Tokyo, Japan; Division of Rheumatology, Department of Internal Medicine, NHO Tokyo Medical Center, Tokyo, Japan; Department of Dentistry and Oral Surgery, Keio University School of Medicine, Tokyo, Japan; Department of Dentistry and Oral Surgery, Keio University School of Medicine, Tokyo, Japan; Division of Rheumatology, Department of Internal Medicine, Keio University School of Medicine, Tokyo, Japan

*Rheumatology* key message

- Distinct autoreactive B-cell clones undergo antigen-driven affinity maturation against multiple autoantigens, collectively recapitulating serum autoreactivity.

Dear Editor, The salivary glands, a major target organ in Sjögren’s disease (SjD), are known to be a site of local production of autoantibodies, including those directed against Ro60, Ro52 and SSB [1, 2]. We previously demonstrated that these antibodies recognize diverse epitopes of their cognate autoantigens and exhibit substantial somatic hypermutation, indicating antigen-driven affinity maturation. We also reported that autoantibodies associated with antisynthetase syndrome, including anti-Jo-1 and anti-EJ antibodies, can be produced within the salivary glands [3]. Here, we report a patient with sicca symptoms who fulfilled the current SjD classification criteria and showed complex serum ANA reactivity, in whom recombinant monoclonal antibodies generated from salivary gland B cells identified three distinct autoantibody specificities.

The clinical characteristics of the patient are summarized in Supplementary Table S1. The patient presented with sicca symptoms, including dry eyes and dry mouth, without other systemic manifestations. Clinical ANA testing revealed speckled, nucleolar and cytoplasmic staining patterns, suggesting the coexistence of multiple autoantibody specificities. Although clinical anti-SSA/Ro testing was positive, it did not differentiate between Ro60 and Ro52 reactivity. Salivary gland biopsy demonstrated lymphocytic infiltration without focus formation. To characterize local B cell responses, we generated 42 recombinant monoclonal antibodies from 48 salivary gland antibody-secreting cells isolated from excess salivary gland biopsy tissue, as previously described [3] (Fig. 1A). We first assessed the recombinant monoclonal antibodies by ANA testing. Whereas serum ANA testing revealed multiple staining patterns (Fig. 1B), analysis of the monoclonal antibodies deconvoluted the composite serum reactivity into nucleolar-, Golgi- and cytoplasmic speckled-pattern antibodies (Fig. 1C–E). Next, we produced recombinant candidate autoantigens corresponding to each ANA pattern, immobilized them on beads and analysed antibody binding by flow cytometry as previously described [3]. Nucleolar-pattern antibodies reacted specifically with RNase P [4], Golgi-pattern antibodies with GOLGA4 [5], whereas Ro52 was recognized by both cytoplasmic speckled-pattern and ANA-negative antibodies (Fig. 1F–H). Overall, 78.6% of salivary gland–derived antibodies were autoreactive, representing multiple autoantibody specificities (Fig. 1I).

**Figure 1:**
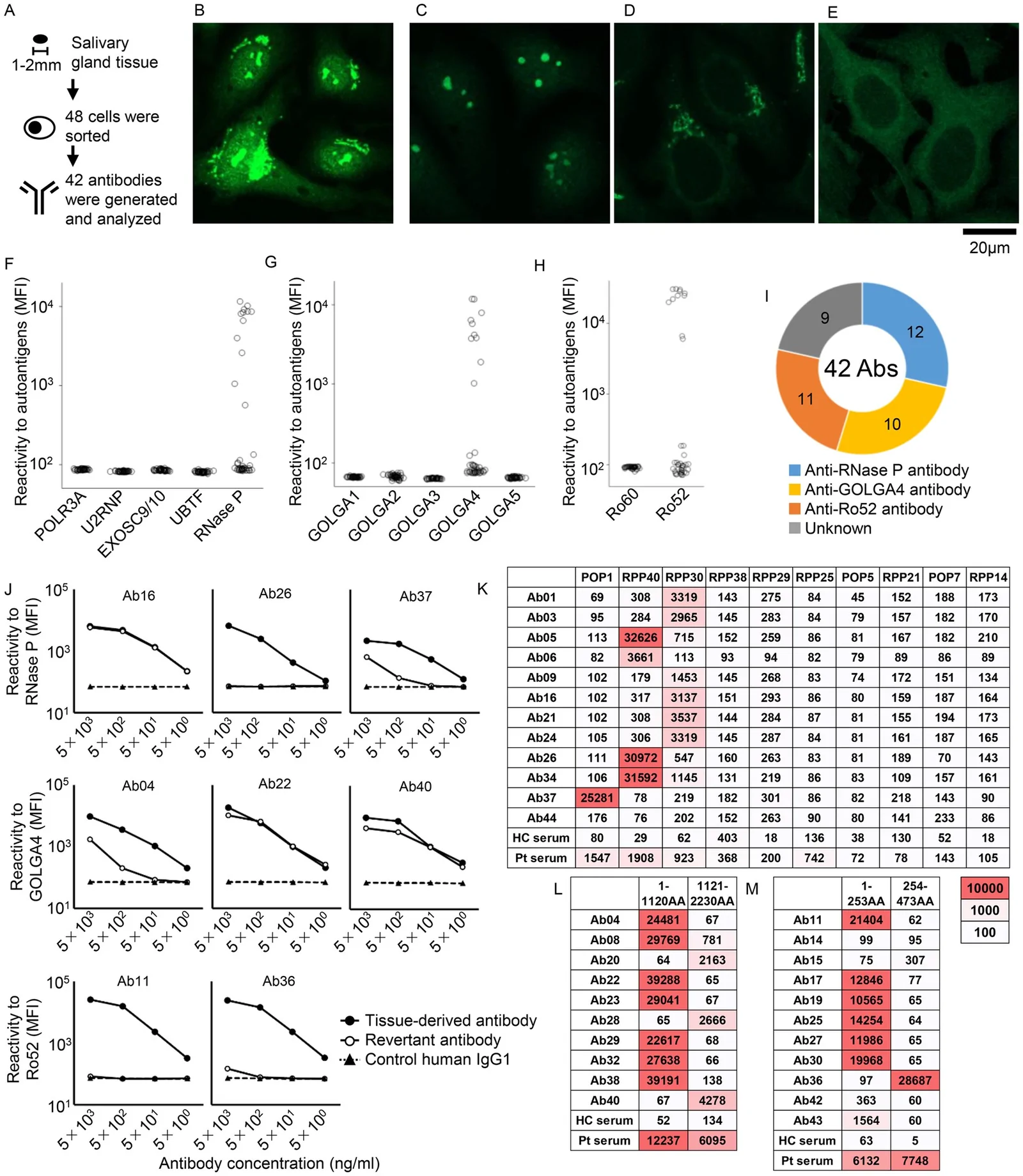
Reactivity of salivary gland–derived autoantibodies with three distinct specificities. (A) Schematic overview of this study. (B) A result of a serum ANA test. (C–E) Representative results of ANA testing observed among the generated monoclonal antibodies: (C) nucleolar pattern, (D) Golgi pattern and (E) cytoplasmic speckled pattern. (F–H) The reactivity of monoclonal antibodies to candidate autoantigen-coupled beads is shown as median fluorescence intensity (MFI): (F) nucleolar pattern, (G) Golgi pattern and (H) speckled pattern. (I) Proportion of each autoantibody specificity among the 42 monoclonal antibodies generated from the disease lesion. (J) Reactivity of representative autoantibodies and their revertant antibodies to the corresponding autoantigen-coupled beads is shown as MFI. (K) Reactivity of each anti–RNase P autoantibody (2 μg/ml) to beads coupled with individual RNase P constituents is shown as MFI. Reactivity of serum (100-fold diluted) from a healthy control (HC) and from this patient (Pt) is also shown. Reactivity of each (L) anti-GOLGA4 and (M) anti-Ro52 autoantibody and serum to beads coupled with the N- and C-terminal fragments of the corresponding autoantigen is shown as MFI

To determine whether these antibodies arose through antigen-driven responses, we analysed immunoglobulin variable-region sequences. Although anti-RNase P antibodies showed modest enrichment of IGHV3-23/IGLV3-21 usage, VH and VL gene usage was otherwise diverse (Supplementary Fig. S1A and B). Comparison with inferred germline sequences revealed substantial somatic hypermutation and amino acid substitutions (Supplementary Fig. S1C and D). To assess the functional contribution of these mutations, germline-reverted antibodies were generated and tested for antigen binding. Germline-reverted antibodies exhibited reduced antigen binding in several cases (Fig. 1J), indicating antigen-driven affinity maturation.

Next, we mapped antigenic targets. We individually immobilized the 10 protein components of the RNase P complex on beads and tested antibody reactivity. Among 12 anti-RNase P antibodies, six recognized RPP30, four recognized RPP40 and one recognized POP1, whereas one failed to bind any individual component, suggesting recognition of a conformational epitope (Fig. 1K). For GOLGA4 and Ro52, N- and C-terminal fragments were analysed separately. Antibodies recognizing both regions were identified for each antigen (Fig. 1L and M), demonstrating broad targeting across the self-antigen molecules. In addition, the recombinant monoclonal antibodies closely recapitulated the serum epitope-binding profile, suggesting that local antigen-specific B cell clones contribute to the polyclonal serum autoantibody response.

These findings demonstrate that analysis of recombinant monoclonal antibodies derived from salivary gland B cells can deconvolute polyclonal serum autoantibody responses into their constituent monoclonal autoantibody specificities. To our knowledge, this is the first demonstration that anti-RNase P and anti-Golgi antibodies can be produced locally within salivary glands. Similar to anti-Ro60 and anti-aminoacyl-tRNA synthetase antibodies [2, 3, 6], these antibodies underwent self-antigen–driven affinity maturation, supporting the notion that antigen-driven maturation of autoreactive B cells is a shared feature of diverse autoimmune responses.

A distinctive feature of this case was the coexistence of multiple autoantibody specificities. Anti-Golgi antibodies have been described in SjD [5], whereas anti-RNase P antibodies are classically associated with SSc [4], and their relationship to salivary gland pathology has not been established. Despite local anti-RNase P antibody production, our patient showed no clinical manifestations of SSc. Whether this finding represents an exceptional feature of SjD or an underrecognized overlap between SjD and SSc-associated immune responses will require further study in additional cases.

In inflammatory myopathies, autoantibody-based classification often better reflects clinical phenotypes than conventional diagnostic categories [7]. Likewise, we recently reported distinct cytokine signatures in anti-SSA and anti-centromere antibody–positive salivary glands [8], supporting a link between autoantibody specificity and local immune pathways. The present findings further suggest that the repertoire of autoantibodies produced within salivary glands may be more diverse than previously appreciated. Future studies defining the molecular pathology associated with individual autoantigen specificities, as well as the interactions between coexisting autoreactive responses, may help clarify the pathogenesis and improve the diagnosis of heterogeneous salivary gland lesions.

## Supplementary Material

keag525_Supplementary_Data

## Data Availability

All unique reagents generated in this study are available with a completed MTA.
