# Supplementary material for: Multiple autoantibody-producing B-cell clones coexist within a single human salivary gland
Source: Rheumatology (Oxford). 2026 Sep 18;65(10):keag525. doi: 10.1093/rheumatology/keag525 (PMC13630444; doi:10.1093/rheumatology/keag525)
Supplement: Supplementary file 1 [file keag525_supplementary_data.pdf]

Supplementary material

Supplementary Figure

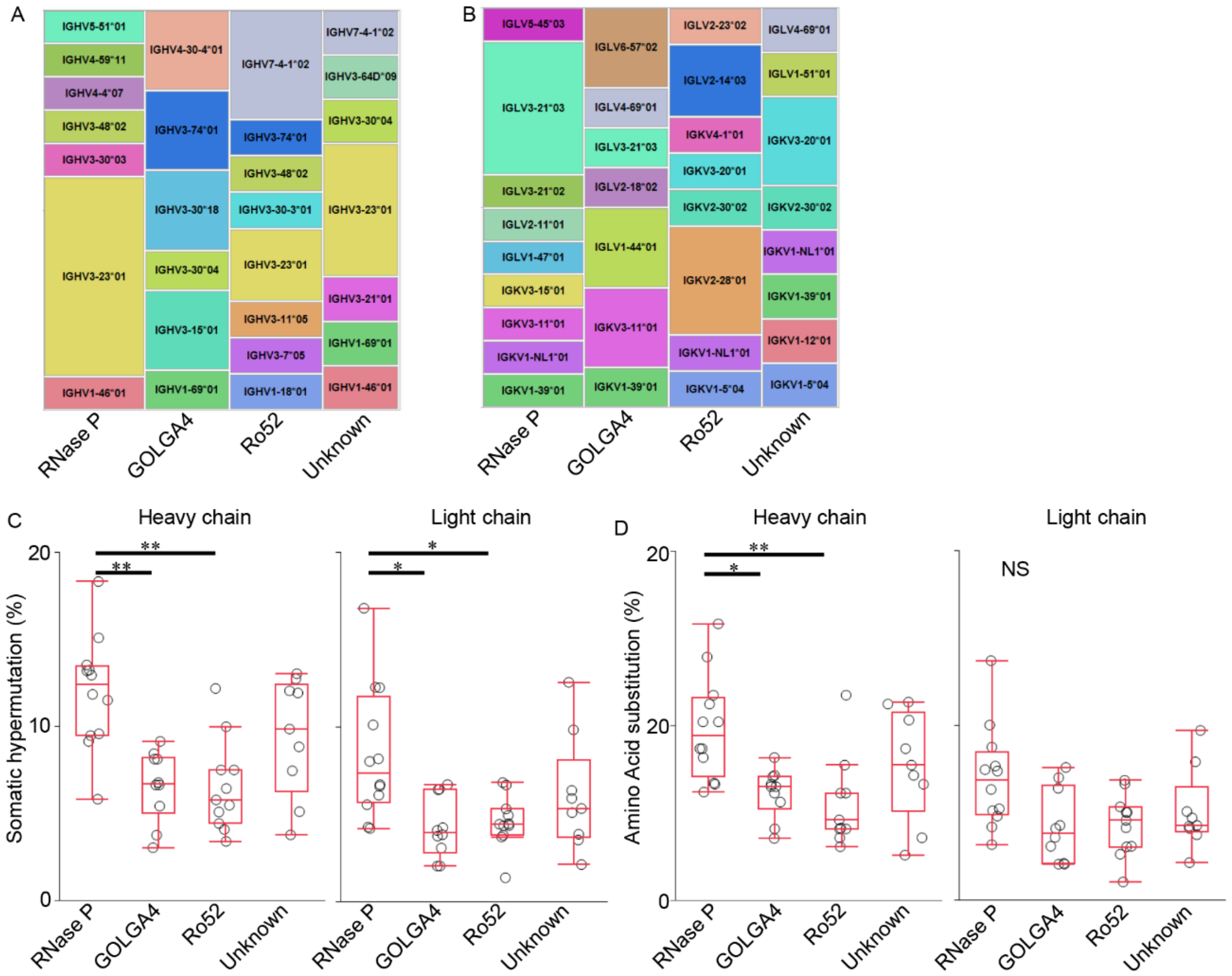

Supplementary Figure S1. Gene usage and mutation frequencies of autoantibodies

Frequency of (A) IgHV and (B) IgLV gene usage among each autoantibody specificity. (C) Number of somatic hypermutations and (D) number of amino acid substitutions according to autoantibody specificity. Steel–Dwass test. *P < 0.05; **P < 0.01.

Supplementary Table

Supplementary Table S1. Patient’s characteristics

Age	71
Sex	Female
2016 ACR/EULAR criteria for Sjogren’s disease	fulfilled
2013 ACR/EULAR criteria for Systemic sclerosis	not fulfilled
Disease duration (months)	36
Medication	-
**Clinical symptoms**	
Dry eye / dry mouse	+ / +
Skin rash	-
Arthritis	-
Interstitial lung disease	-
Skin thickening	-
Fingertip lesions	-
Telangiectasia	-
Abnormal nailfold capillaries	-
Raynaud's phenomenon	-
Lymphadenopathy	-
Renal involvement	-
**Laboratory tests**	
Rheumatoid factor	<5
ANA titre, pattern	1:40, speckled<br>1:80, nucleolar<br>1:320, Cytoplasmic<br>(Golgi, speckled)
Serum anti-SSA/Ro antibody	>240
Serum anti-SSB/La antibody	negative
Serum anti-Scl-70 antibody	negative
Serum anti-centromere antibody	negative

Serum anti-RNA polymerase III antibody	negative
IgG (mg/dl; normal range 870-1700)	1437
C3 (mg/dl; normal range 65-135)	83
C4 (mg/dl; normal range 13-35)	34
C-reactive protein (mg/dl; normal range 0-0.35)	0.01
Free T3 (pg/ml; normal range 2.0-4.5)	2.6
Free T4 (ng/dl; normal range 0.7-1.8)	1.2
Thyroid-stimulating hormone (μIU/ml; normal range 0.3-4.5)	1.62
**Diagnostic tests**	
Salivary flow (ml/10 min)	0.9
Salivary gland focus score	0[a]
Schirmer test (ml/5 min)	ND
Superficial punctate keratitis	ND
Salivary gland scintigraphy	
Parotid glands uptake (R/L, %; normal range > 0.25%)	0.12 / 0.11
Parotid glands excretion (R/L, %; normal range > 50%)	6.02 / 13.26
Submandibular glands uptake (R/L, %; normal range > 0.15%)	0.07 / 0.07
Submandibular glands excretion (R/L, %; normal range > 50%)	1.06 / 6.25

[a]Periductal and interacinar mononuclear cell infiltration including plasma cells, without focal lymphocytic sialadenitis.

ND, not determined
